# Synthetic nucleic acids in a post-agent biosecurity Era

**DOI:** 10.3389/fbioe.2026.1819026

**Published:** 2026-04-20

**Authors:** Jacob Hofman-Bang, Katrine Finderup Nielsen, Stine Juhl, Cyril Jean-Marie Martel

**Affiliations:** Center for Biosecurity and Biopreparedness, Statens Serum Institut, Copenhagen, Denmark

**Keywords:** biological weapons, biosecurity, DNA synthesis, dual-use, screening

## Introduction

**FIGURE 1:**
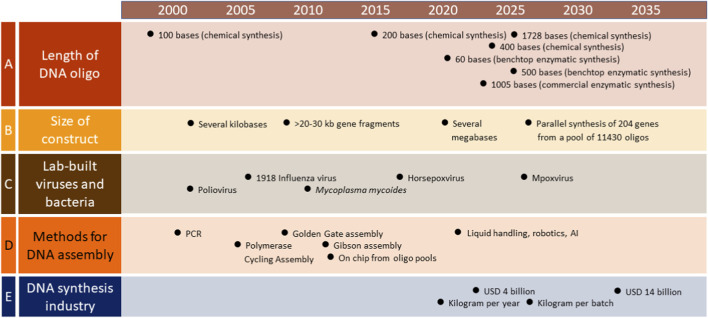
Progression in nucleic acid synthesis technology. **(A)** describes the increases in the length of DNA oligonucleotides that can be synthesized either by chemical phosphoramidite or enzymatic synthesis, commercially or by benchtop devices. **(B)** describes the size of constructs obtainable by assembling synthetic DNA fragments, from a single poliovirus genome to a bacterial genome to the parallel synthesis of hundreds of genes from a pool of oligonucleotides. **(C)** shows examples of synthesis of functional viruses and bacteria. **(D)** shows the development of techniques employed to assemble DNA oligonucleotides into larger gene fragments. **(E)** shows the increase in the estimated worldwide DNA synthesis capacity and market size in US dollars.

Since the 1980s, DNA synthesis technology has evolved from chemical to enzymatic methods, from single-column synthesis to massively parallel chip-based approaches, and from a handful of commercial providers to a vast global market supplemented by decentralized benchtop systems (Figure 1). These advances have dramatically reduced costs while increasing the length of synthesizable oligonucleotides, from a few hundred bases in 2015 to well over a thousand in 2026. Longer oligonucleotides, combined with improved assembly methods, have made the construction of large genetic constructs both easier and more reliable (Wan et al., 2017). This progress is reflected in the size and complexity of viruses synthesized in the laboratory, ranging from the relatively small 7.5 kb poliovirus in 2002, which required more than 2 years of painstaking work (Cello et al., 2002), to the 212 kb horsepoxvirus in 2017, reportedly completed in less than 6 months at a cost of approximately USD 100,000 (Noyce et al., 2018). While these developments have delivered substantial benefits in fields such as cancer medicine, vaccine development, and plant biology, they have also raised concerns about dual-use risks that existing biosecurity frameworks may not adequately address (NSABB, 2006).

### Current biosecurity frameworks and legacy biological weapons programs

Contemporary biosecurity frameworks reflect lessons drawn from twentieth-century bioweapons programs, when states pursued large-scale efforts to weaponize a relatively limited set of high-consequence agents, typically in highly specialized facilities (Carus, 2017). Governance was therefore built around a threat model centered on identifiable, dangerous pathogens and the infrastructure required to handle, cultivate, and disseminate them. This legacy remains visible in international and national legal instruments: the Biological Weapons Convention prohibits the development and stockpiling of biological weapons but leaves implementation largely to national authorities, and many states operationalize their obligations through select agent lists and licensing regimes tied to those lists. Export control arrangements similarly reflect a list-centric logic. The Australia Group, for example, harmonizes controls on pathogens, toxins, and dual-use equipment categories, and historical relevance to bioweapons programs has been an important consideration in decisions about list inclusion (Shaw, 2016). Denmark’s national biosecurity program follows this model by using the Australia Group list as the basis for regulated organisms and materials (Steenhard, 2015). Under Danish law, entities that handle listed agents must comply with licensing, reporting, and security requirements, thereby complying with a list-based control strategy.

The logic behind these systems is straightforward: restricting access to a defined set of hazardous biological materials, and to the tools required to weaponize them at industrial scale, reduces the likelihood of catastrophic misuse. Historical cases underscore why these constraints have mattered: Iraq’s biological weapons program in the 1980s and 1990s succeeded in producing bulk agent but struggled with reliable dissemination (Smithson, 2025); converting material into an effective aerosol weapon proved technically demanding, underscoring that possession alone was insufficient. Likewise, Aum Shinrikyo sought to acquire virulent strains and pursue biological attacks in the early 1990s but repeatedly failed to obtain and effectively use high-consequence pathogens, despite substantial resources and scientific training among its members (Danzig et al., 2011). These episodes illustrate the preventive value of controls on both pathogens and specialized equipment.

Tacit expertise has provided an additional layer of protection (Revill and Jefferson, 2014). Even where relevant scientific literature is accessible, achieving effective stabilization and dissemination of biological agents has typically required substantial time, resources and experience. In practice, this kind of know-how could not be easily acquired; it tended to be developed through sustained, programmatic effort. In that context, list-based regimes have limited proliferation by targeting critical inputs and leveraging the intrinsic difficulty of turning biological materials into reliable weapons.

### Dual-use risks have gotten more diffuse

A growing consensus in the biosecurity community is that advances in DNA synthesis (Liu et al., 2025), laboratory automation (Liu et al., 2025), computational design (Mishra et al., 2026), and the convergence of biology with chemistry and nanotechnology (Wang et al., 2024) have fundamentally altered the assumptions behind list-based approaches. These developments have expanded what is technically feasible beyond traditional state programs. Capabilities such as gene sequencing, gene editing, and *de novo* DNA synthesis, once confined to elite facilities, are increasingly embedded in commercial platforms and academic laboratories, sometimes with the help of artificial intelligence (Li et al., 2026). This democratization of biotechnology (Byagathvalli et al., 2021) empowers scientists and innovators who would otherwise be excluded from combating global challenges because of economic or infrastructure barriers, and this positive trend is by no means driven by weaponization purposes. However, it does also result in a wider range of pathways through which biological functions can be engineered, including routes that can, at least in principle, mitigate or bypass constraints that have historically limited weaponization.

Within this broader shift, synthetic biology has been a particular focus of scrutiny because it enables the systematic modification of biological organisms at the genetic level. Gene editing tools such as CRISPR-based systems, synthetic genomics (Sleator, 2010), and computationally enabled protein design (Johansen et al., 2025) have drastically changed the way scientists explore and alter biological functions. Design–build-test cycles that integrate software, automated synthesis, and high-throughput screening (He et al., 2026) have accelerated experimentation and reduced reliance on slow, highly contingent trial-and-error approaches. As a result, dual-use potential is no longer confined to rare pathogens or specialized biodefense programs: it increasingly arises within mainstream life science research and industrial biotechnology. The same platforms that enable vaccine development, enzyme engineering, or sustainable materials research can be redirected toward harmful ends. The risk of misuse is therefore more diffuse, being distributed across widely accessible capabilities rather than concentrated in a small number of tightly controlled organisms.

From a regulatory perspective, these developments weaken regimes focused primarily on lists of pathogens and equipment. As alternative methods to achieve weaponization proliferate, restrictions on tangible items get easier to circumvent. At the same time, the ubiquity of dual-use tools, combined with the substantial benefits of legitimate applications, makes imposing restrictions on broad swaths of biotechnology practically challenging, ethically contested, and politically unpalatable. One notable exception is synthetic nucleic acids: nucleic acid synthesis, whether conducted through commercial providers or benchtop systems, is a critical step in many modern design–build–test pipelines. Regulations that meaningfully shape this process can therefore have a high preventive value against the misuse of synthetic biology.

### Synthetic nucleic acids: the new chokepoint?

Synthetic nucleic acids are chemically or enzymatically produced strands of DNA or RNA created from digital sequence information rather than copied from living organisms. Once a sequence is designed *in silico*, it can be ordered, assembled, and expressed to produce proteins, regulatory elements, entire pathways, or large genomic constructs (Cleij et al., 2025). Historically, synthesis relied on phosphoramidite chemistry. While robust, the process is stepwise and accumulates errors, limiting oligonucleotide lengths and requiring multi-fragment assembly to obtain larger constructs. This has imposed a practical constraint: even if short sequences have been readily accessible, building longer genes or genomes has demanded time, expertise, and resources, which has raised the threshold for misuse. However, this constraint is weakening: enzymatic DNA synthesis approaches and improvements in assembly methods have simplified construction at gene and viral-genome scales, while commercial offerings increasingly include longer fragments, higher quality and complex constructs. The scientific community today has access to automated systems that can synthesize DNA fragments up to 750 bases, which is long enough to code for small toxic proteins, and experts foresee the automated DNA technology to mature to produce DNA fragments of up to 7000 bases (Carter et al., 2023). Automation (Kim et al., 2025) and improved design software (Shor et al., 2025) reduce the burden of assembling multi-part systems, and manufacturing feedback tools can identify problematic sequence features and propose design adjustments (Yu et al., 2024). In addition, other technologies that are not directly related to the synthesis of nucleic acids or their assembly, but are critical for downstream applications are also evolving rapidly, and becoming cheaper and easier to use: for example, designing artificial operons (Basitta et al., 2017) that code for all enzymes or proteins necessary to obtain a specific product, or producing large amounts of plasmids for large-scale dissemination (Eurogentec, 2024).

Although the discussion about the misuse potential of synthetic nucleic acids often revolves around the purported ability to artificially produce existing restricted biological agents such as viruses or toxins, there is actually a much broader range of applications that have a misuse potential: DNA/RNA origami (Krissanaprasit et al., 2024), DNA-based artifical receptors (Chen et al., 2023), plasmids, self-replicating RNA (Wagner and Mutschler, 2023), short hairpin RNA (Peretz et al., 2018), microRNAs (Wang et al., 2026), small interfering RNAs (Serra et al., 2025), guide RNA (Lim and Lee, 2024), locked nucleic acid gapmers (Dauksaite et al., 2023), xeno nucleic acids (Murayama and Asanuma, 2021), circular RNA (Mellor et al., 2026), and aptamers (Zhou et al., 2026). These constructs can be misused in a variety of ways. First, synthetic DNA can be assembled to recreate existing or extinct viruses that an actor would not otherwise have access to (Chen J. et al., 2025). Proof-of-concept work has shown that certain viral genomes and bacteriophages can be synthesized from sequence information and recovered in laboratory settings (Levrier et al., 2024; Brixi et al., 2025). While such efforts may have legitimate scientific aims, they demonstrate that sequence access combined to synthesis capacity can, in principle, substitute access to natural samples (Oldfield et al., 2017; Vashee et al., 2017; Cao et al., 2023; Spatz et al., 2023). Additionally, existing pathogens can be modified to enhance their pathogenicity or escape pre-existing immunity in the target population. Second, nucleic acids can act as effectors when delivered into cells, without needing to be translated into proteins (Bloom et al., 2021; Shin et al., 2022). Therapeutic modalities that regulate gene expression, alter transcriptional or translational processes, or reshape a cellular state are increasingly mature (Lu et al., 2026). The same principles could, in misuse contexts, be applied to disrupt normal cellular function or modulate physiological pathways in harmful directions. This is particularly true if paired with effective delivery systems such as nanoparticles (Bai and Su, 2025), which have also become easier to customize and produce at scale (Hu et al., 2025). Third, nucleic acids can encode biologically active molecules. Through *in vivo* cellular expression or through cell-free production, synthetic sequences can be used to express proteins or peptides at scale (Chen H. et al., 2025). In legitimate settings, this enables the production of vaccines, enzymes, and industrial biocatalysts; however, the dual-use concern is that encoded products could also include effectors with harmful biological activity. Fourth, programmable gene-editing systems can allow potentially harmful interventions on specific genetic targets. CRISPR-related tools can enable precise modification of DNA or RNA and, in some cases, epigenetic states (Nakamura et al., 2021). While transformative for medicine and agriculture, they also illustrate the theoretical ability to target a wide range of genomic loci, raising concerns about interference with biological functions (Davies, 2019). Gene drives (Bier, 2022), for example, have been the object of much discussion for the risks they might present.

Other technological breakthroughs reinforce the idea that synthetic nucleic acids can be misused in a plethora of ways, potentially paving the way for biological weapons that are more potent, more reliable, more flexible, and harder to detect than living organisms and their toxins: computational protein design expands the repertoire of novel molecules that can bind receptors in target cells (Durojaye et al., 2025; Fox et al., 2025); nanotechnology broadens delivery and assembly possibilities (Fanarraga and García Hevia, 2026); engineered nucleic-acid structures illustrate how genetic material can be used for sensing, signaling, and cargo transport (Samanta et al., 2026).

## Discussion

The promising potential of synthetic nucleic acids as a chokepoint around which to organize new biosecurity regimes has not gone unnoticed. The UK, the US, New Zealand and the EU have all put forward guidance and legislations introducing frameworks for the systematic screening of synthetic nucleic acid orders. Various actors are working on technical solutions and standards to facilitate their implementation, and several reports are pointing out the cost-effectiveness of such measures. However commendable, these efforts are still very much focused on an agent-centric approach, where orders are screened against databases of sequences from pathogens of interest. This approach has two issues: first, current screening tools from synthetic nucleic acid providers can be bypassed, as shown recently (Wittmann et al., 2025): for example, by usingprotein folding prediction algorithms to design proteins (Zhou et al., 2020) with similar 3D structures and properties as proteins from biological agents of concern, but with very distinct nucleic acid and amino acid sequences. A caveat is that only some of these novel proteins have had their function validated *in vitro*. Second, as discussed previously, there is in theory a multitude of constructs that could be built using synthetic nucleic acids as effectors without relying on a sequence from a pathogen. Mitigating both these issues will likely require using the very same technology that already threatens to undermine sequence screening: AI-powered tools able to predict the structure, function and effect of unknown sequences in a wide range of contexts and scenarios, thereby identifying dangerous sequences even if they are not part of a previously existing database of sequences of concern. The dilemma is that such tools will themselves have a high potential for misuse, and their development, training and governance should therefore follow the core principles of AI alignment (Ji et al., 2025) to guarantee that they actually minimize biosecurity risks rather than increase them.

Future biosecurity regimes will need to address the increased diffuseness of dual-use risks, and combining traditional list-based approaches with new controls on nucleic acid synthesis is only part of the answer. Early and sustained engagement with researchers within a very broad range of disciplines, giving them tools and guidance to empower them to protect their own work from misuse will also be a critical step.
